# Glycemic Variability as a Predictor of Mortality in Sepsis Patients With Concurrent Persistent Inflammation, Immunosuppression, and Catabolism Syndrome

**DOI:** 10.1002/iid3.70400

**Published:** 2026-03-06

**Authors:** Shuhang Wang, Li Liu, Bowen Li, Yancun Liu, Yanfen Chai

**Affiliations:** ^1^ Tianjin Medical University General Hospital Tianjin China; ^2^ College of Environmental Science and Engineering Nankai University Tianjin China

**Keywords:** Boruta algorithm, glucose variability coefficient, machine learning, persistent inflammation, immunosuppression, and catabolism syndrome, sepsis

## Abstract

**Background:**

Sepsis is a life‐threatening condition caused by infection, which triggers dysregulated systemic inflammatory responses. Among sepsis patients, those who concurrently develop persistent inflammation, immunosuppression, and catabolism syndrome (PICS) have a significantly poorer prognosis. It has been demonstrated that associations exist between elevated glycemic variation coefficient (GVC) levels and the development of PICS in septic populations. However, the association between GVC and adverse clinical outcomes in the subgroup of septic patients with PICS requires further investigation.

**Methods:**

The study analyzed data from the Medical Information Mart for Intensive Care IV (MIMIC‐IV) database, which comprised 1353 critically ill septic patients who developed nosocomial infections during hospitalization. Based on the 2024 Critical Care Medicine Guidelines on Glycemic Control, the patients included in this study will be divided into GVC < 20 group, 20 ≤ GVC ≤ 36 group, and GVC > 36 group. The primary outcome measure was 28‐day all‐cause mortality, with secondary outcomes comprising in‐hospital mortality and 180‐day mortality. Cox proportional hazards regression and Kaplan–Meier analysis were utilized to examine the relationship between GVC and adverse outcomes. The Boruta algorithm evaluated the predictive capacity of GVC, followed by the development of prognostic models through machine learning (ML) and deep learning (DL) algorithms, externally validated using an independent cohort of 116 patients from the Emergency Department of Tianjin Medical University General Hospital.

**Results:**

The analysis included 1353 septic patients. Kaplan–Meier analysis indicates that the highest GVC tertile has significant differences in 28‐day and 180‐day mortality rates. Cox regression analysis revealed that patients in the highest GVC tertile had a significantly elevated 28‐day mortality risk. (OR = 1.60, 95% CI: 1.11–2.32, *p* < 0.05). The Boruta algorithm identified GVC as a key predictor for mortality risk. The 28‐day mortality prediction model developed using tabular prior‐data fitted network (TabPFN) achieved an area under the curve (AUC) of 0.960.

**Conclusion:**

GVC demonstrated significant correlations with 28‐day and 180‐day mortality in sepsis patients complicated by PICS. DL models confirm the utility of GVC as a robust prediction tool for septic patients, providing valuable references for clinical decision‐making.

## 1 Introduction

Severe sepsis has garnered significant global attention due to its escalating incidence and persistently high mortality rates, with patient mortality rates reaching up to 53.4% [1]. Sepsis involves highly intricate pathophysiological processes, with its inherent heterogeneity necessitating classification of patients into clinically distinct subphenotypes. While advances in critical care technology and sepsis management have improved early survival rates, a significant proportion of patients progress to chronic critical illness (CCI) [2]. Patients developing Persistent Inflammation, Immunosuppression, and Catabolism Syndrome (PICS), featured by sustained inflammation, immunosuppression, and catabolic metabolism, show significantly worse long‐term survival than rapid‐recovery patients. This disparity stems from pathological homeostatic derangement driving progressive functional decline [3, 4, 5].

Current studies focused on biomarkers, clinical characteristics, and their associations with outcomes in sepsis. Frugoli et al. confirmed significant correlations between glycemic levels/fluctuations and clinical outcomes in septic patients [6]. Several studies suggested that glycemic variability may serve as an indicator of inflammatory status and metabolic dysregulation in patients and potentially contribute to prognostic assessments for septic patients [7, 8, 9]. Elevated glycemic variability may strongly predict compromised homeostatic regulation in patients. Strategic management of both stress‐induced hyperglycemia and hypoglycemia is clinically recognized as a critical factor in improving survival rates among sepsis patients [10, 11]. Current evidence identified glycemic variation coefficient (GVC) as a significant risk factor for PICS development in sepsis patients [12]. However, the further relationship between GVC and mortality in sepsis patients complicated by PICS remains insufficiently characterized.

This study employs a retrospective cohort design, integrating advanced machine learning and deep learning techniques to extract clinical data from the MIMIC‐IV database for mortality prediction modeling. This study aims to develop a GVC‐based prediction model for mortality risk in sepsis patients with PICS, providing clinicians with a precise prognostic assessment tool. This will optimize management strategies and treatment approaches for septic patients complicated by PICS, ultimately aiming to improve clinical outcomes and health‐related quality of life.

## 2 Materials and Methods

### 2.1 Data Collection

This study utilizes data extracted from the MIMIC‐IV v2.2 database. As one of the largest publicly accessible critical care repositories, it comprises electronic health records from > 50,000 patients admitted to the intensive care units (ICU) at Beth Israel Deaconess Medical Center (BIDMC) in Boston, Massachusetts, between 2008 and 2019 [13]. The Institutional Review Board of BIDMC granted a waiver of informed consent and authorized data sharing for research purposes. The author (Li Liu) has obtained access privileges to this database (Certification ID: NO. 61529195). The prediction model was developed using the MIMIC‐IV database and underwent external validation with patient data from the Emergency Medicine Department of Tianjin Medical University General Hospital.

### 2.2 Patients

**Figure 1:**
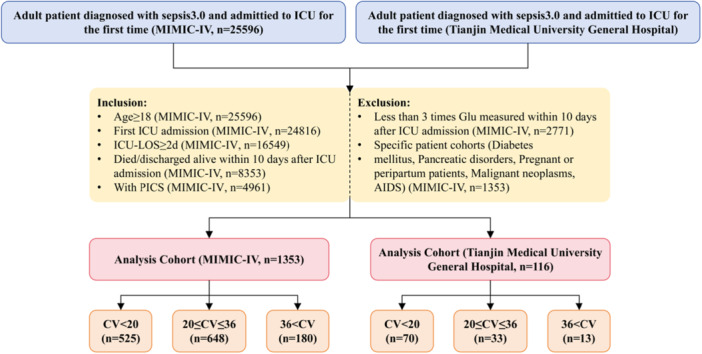
Study flowchart for selecting the study population from the MIMIC‐IV database and Tianjin Medical University General Hospital.

The MIMIC‐IV database yielded an initial extraction of 25,596 patients. Application of pre‐specified inclusion/exclusion criteria established a final analytical cohort of 1353 patients. This study additionally enrolled 116 eligible patients meeting predefined inclusion criteria at Tianjin Medical University General Hospital between January 2022 and December 2023 from electronic health records (Figure 1). Inclusion criteria included: (1) age ≥ 18 years; (2) sepsis diagnosis per Sepsis‐3 criteria (confirmed infection + Sequential organ failure assessment (SOFA) score ≥ 2) [14]; (3) ICU length of stay > 48 h; (4) ≥ 3 venous blood glucose measurements during ICU admission; (5) exclusion of patients who died or were discharged within 10 days of ICU admission; and (6) PICS patients: CRP > 3.0 mg/L, lymphocyte count < 800/μL or albumin < 3.0 g/dL after 10 days of ICU admission [4, 15, 16]. Exclusion criteria included: (1) conditions potentially confounding glycemic metabolism, including pre‐existing diabetes mellitus (Type 1 or 2), pancreatic disorders (acute/chronic pancreatitis or pancreatectomy); (2) missing SOFA score components essential for sepsis diagnosis, indeterminate 28‐day survival status, insufficient glucose measurements (< 3) or non‐consecutive sampling (> 72‐h gaps between tests); and (3) special populations, including Pregnancy/peripartum patients, active malignancies, AIDS, and pregnant individuals (redundancy explicitly retained per protocol) [17]. The primary outcome was selected as 28‐day mortality, with secondary outcomes, including in‐hospital mortality and 180‐day mortality among patients.

### 2.3 Data Extraction

This study employed the pgAdmin tool (v4.30) to extract core demographic characteristics, including patient age, gender, and admission body weight. Comorbidity data, including heart failure, hypertension, and malignancies, were extracted using International Classification of Diseases (ICD‐10‐CM) coding standards aligned with study‐specific requirements. Vital signs, including heart rate, systolic blood pressure, respiratory rate, and arterial oxygen saturation, were concurrently monitored. Laboratory assessments included: hematological indices (red blood cells, white blood cells, platelets, lymphocytes, neutrophils, and monocytes), electrolyte and acid–base balance parameters (serum sodium, potassium, calcium, anion gap, pH, partial pressure of carbon dioxide, arterial partial pressure of oxygen, lactate, and blood glucose), coagulation function (prothrombin time (PT), partial thromboplastin time (PTT), and international normalized ratio (INR)), and hepato‐renal biomarkers (total bilirubin, aspartate aminotransferase (AST), blood urea nitrogen (BUN), and serum creatinine). SOFA scores, Charlson comorbidity index, Glasgow coma scale (GCS) scores, and simplified acute physiology Score II (SAPS II) were concurrently documented to quantify disease severity. The GVC was calculated using the formula:

(1) $$\mathrm{GVC}\;(\%)=\frac{\mathrm{SD}}{\bar{x}}\times 100\%$$

where SD represents the dispersion of blood glucose measurements around the arithmetic mean glucose concentration, $\bar{x}$ represents the arithmetic mean blood glucose concentration.

### 2.4 Statistical Analysis

We stratified patients into three distinct risk cohorts based on predefined GVC thresholds in this retrospective study, which did not involve a prior sample size calculation [18]. The multiple imputation was employed to handle missing values. Normally distributed continuous variables were expressed as mean (standard deviation) and compared using analysis of variance, while non‐normally distributed variables were analyzed with Mann–Whitney *U* or Kruskal–Wallis tests. Categorical variables were described as counts (percentages) and evaluated with Chi‐square (*χ*²) or Fisher's exact tests.

Survival analysis was performed using Kaplan–Meier curves to estimate 28‐day and 180‐day all‐cause mortality rates for three GVC groups. Using the Log‐rank test for trend to evaluate the differences in survival curves. The MIMIC database records at least 1 year of follow‐up of patients outside the hospital. For lost patients, the final confirmed survival date was reviewed, and only 28 days or 180 days of all‐cause death were considered as outcome events. The Cox proportional hazards regression model was used to analyze the in‐hospital mortality rate, 28‐day mortality rate, 180‐day mortality rate, hazard ratios (HRs), and their 95% confidence intervals (95% CI). Model I remained unadjusted; Model II was adjusted for age, weight, gender, heart rate, respiratory rate, systolic blood pressure, SOFA score, and absolute lymphocyte count. Using Schoenfeld residuals to rigorously test the proportional hazards hypothesis of the Cox model. When the proportional hazards assumption was found to be statistically significant, we employed stratified Cox model strategy to account for the time‐varying effects of the covariates (Supporting Information: Figure S1). In addition, we used age as the primary outcome as a positive control to ensure the robustness of this study, and the results showed that as age increased, the 28‐day mortality rate of patients also increased (HR = 1.022, 95% CI: 1.014–1.031, *p* < 0.001). This is consistent with the results of previous studies [19]. All statistical analyses were performed using GraphPad Prism version 10 and R version 4.4.1. The threshold for statistical significance was set at *p* < 0.05.

### 2.5 Modeling Methodology

The Boruta algorithm calculates the weight of each feature variable in the prediction results and sorts them, dividing them into three categories based on their importance: “confirmed,” “tentative,” and “excluded.” The variables marked as “confirmed” are considered the most influential and are included in subsequent ML models for in‐depth analysis. In this study, the Boruta function explicitly set the key iteration parameter maxRuns = 500, which means that the random forest model was iteratively trained 500 times to ensure stable feature importance estimation. The imputed dataset was randomly divided into the training cohort (80% of data) and the test cohort (20% of data). The synthetic minority over‐sampling technique (SMOTE) was conducted to generate synthetic samples to reduce class imbalance in the raw training cohort to enhance the robustness in prediction and extrapolation [20]. Furthermore, all data values of continuous features were normalized to the range of 0–1 according to Equation 2 to eliminate dimensional differences and fasten the learning process [21]. The categorical features were converted into numerical variables by label encoding to unify the data format [22].

(2) $$x=\frac{x_{\mathrm{raw}}-x_{\min}}{x_{\max}-x_{\min}}$$

where x is the normalized value; $x_{\mathrm{raw}}$ is the raw value that has not been normalized; $x_{\min}$ and $x_{\max}$ are the minimum and the maximum values in the raw dataset, respectively.

Six ML models, including COX, random forest (RF), extreme gradient boosting (XGBoost), light gradient boosting machine (LightGBM), categorical boosting (CatBoost) and natural gradient boosting (NGBoost), and two DL models, including artificial neural network and tabular prior‐data fitted network (TabPFN), were selected as candidate models to predict the 28‐day mortality risk in critically ill sepsis patients. The grid search was performed in the training cohort to achieve the optimal hyperparameters for each model and the results are shown in Supporting Information: Table S1. The prediction performance of trained models was evaluated using accuracy, precision, recall, F1 score and the area under the receiver operating characteristic (ROC) curve (AUC). In addition, model calibration was evaluated by comparing observed probabilities vs. predicted probabilities [23]. Decision curve analysis (DCA) was employed to evaluate the clinical utility of the proposed multimodal model in assessing mortality risk among sepsis patients with PICS subpopulations [24].

All models were coded using Python 3.10. ML models were imported from Sklearn and its corresponding Python library [25, 26, 27, 28, 29]. DL models were implemented by TensorFlow and PyTorch [30, 31]. Pandas and Numpy were used to support the development of the data processing pipeline.

### 2.6 Model Interpretation

The Shapley Additive exPlanation (SHAP) method was adopted to interpret the final model. SHAP connects optimal credit allocation with local explanations using the classical Shapley values from game theory and their related extensions, which represent a thorough theoretical demonstration of consistent and unbiased interpretation methods [22, 23]. The Shapley value $\phi_{i}$ of the feature i was calculated according to Equation 3 [24]. The Shapley value of each feature quantified its contribution, whether negative or positive. A feature with a higher mean absolute Shapley value implied a greater impact on the model output.

(3) $$\phi_{i}=\sum_{S\subseteq N∕\left\{i\right\}}\frac{\left|S\right|!\left(n-\left|S\right|-1\right)!}{n!}\left(f\left(S⋂\left\{i\right\}\right)-f\left(S\right)\right)$$

where S is the subsets of all features with feature i; f(S∩{i}) denotes the prediction by the established EL model considering feature i; and f(S) is the prediction without considering feature i. The differences among all possible subsets of S⊆n are calculated due to the dependency of the effect of withholding a feature on other features in the model.

## 3 Results

### 3.1 Baseline Features

Clinical data were extracted for 1353 sepsis patients with PICS from the MIMIC‐IV database and 116 patients from the Emergency Medicine Department of Tianjin Medical University General Hospital (Figure 1). Table 1 summarizes patient baseline characteristics: Males comprised 773 cases (52%), with 215 (15.89%) having myocardial infarction, 399 (29.50%) heart failure, and 229 (16.93%) cerebrovascular disease. Participants were stratified into three cohorts by GVC risk thresholds: Group 1 (GVC < 20, *n* = 525), Group 2 (20 ≤ GVC ≤ 36, *n* = 648), and Group 3 (GVC > 36, *n* = 180). Group 3 demonstrated elevated platelet count, glucose, serum calcium, anion gap, serum creatinine, lactate levels, SOFA scores, and SAPS II scores. The basic characteristics of 116 patients in the emergency department of Tianjin Medical University General Hospital are shown in Supporting Information: Table S2.

**Table 1:** Baseline characteristics of patients stratified by GVC.

Features	Group 1 (GVC < 20)	Group 2 (20 ≤ GVC ≤ 36)	Group 3 (GVC > 36)	*p*‐value
Demographic				
Gender (male) *n* (%)	319 (60.76)	357 (55.09)	97 (53.89)	0.096
Height (cm) (median (IQR))	171.99 (163.00, 178.00)	168.00 (161.20, 175.17)	168.00 (160.00, 175.00)	0.003
Weight (kg) (median (IQR))	79.30 (67.05, 98.00)	78.30 (67.73, 95.40)	73.28 (60.00, 88.83)	< 0.001
Age (years) (median (IQR))	61.84 (48.73, 74.40)	64.13 (53.47, 75.20)	62.37 (51.69, 75.05)	0.062
Comorbidities				
Myocardial infarct *n* (%)	75 (14.29)	111 (17.13)	30 (16.70)	0.415
Congestive heart failure *n* (%)	132 (25.14)	206 (31.79)	62 (34.40)	0.018
Peripheral vascular disease *n* (%)	59 (11.23)	96 (14.81)	27 (15.00)	0.164
Cerebrovascular disease *n* (%)	112 (21.33)	94 (14.51)	24 (13.33)	0.002
Dementia *n* (%)	21 (4.00)	22 (3.40)	14 (7.78)	0.073
Chronic pulmonary disease *n* (%)	150 (28.57)	170 (26.23)	39 (21.67)	0.033
Rheumatic disease *n* (%)	18 (3.43)	42 (6.48)	5 (2.78)	0.021
Peptic ulcer disease *n* (%)	22 (4.19)	35 (5.40)	9 (5.00)	0.61
Paraplegia *n* (%)	49 (9.33)	44 (6.79)	9 (5.00)	0.1
Renal disease *n* (%)	86 (16.38)	141 (21.76)	40 (22.20)	0.047
Charlson comorbidity index (mean (SD))	4.06 ± 2.52	4.50 ± 2.44	4.60 ± 2.54	0.005
Clinical indicators				
Hemoglobin (g/dL) (median (IQR))	10.10 (8.68, 11.80)	9.85 (8.75, 11.40)	9.87 (8.67, 11.12)	0.343
Hematocrit (%) (median (IQR))	30.50 (26.56, 35.85)	29.85 (26.82, 34.32)	29.52 (26.44, 34.35)	0.385
Platelet (10^9/L) (median (IQR))	160.20 (105.20, 236.7)	151.77 (100.35, 223.8)	125.00 (86.22, 208.50)	0.015
WBC (10^9/L) (median (IQR))	11.85 (8.43, 16.50)	12.61 (8.40, 17.60)	11.62 (7.73, 17.83)	0.447
RBC (10^12/L) (median (IQR))	3.34 (2.88, 3.88)	3.31 (2.88, 3.81)	3.27 (2.90, 3.75)	0.643
Creatinine (mg/dL) (median (IQR))	1.08 (0.75, 1.93)	1.25 (0.80, 2.10)	1.38 (0.88, 2.18)	0.019
BUN (mg/dL) (median (IQR))	24.00 (14.50, 39.42)	25.63 (15.00, 42.30)	27.00 (13.08, 43.63)	0.631
Glucose (mg/dL) (median (IQR))	120.00 (105.88, 136.13)	128.33 (103.83, 157.31)	140.00 (111.50, 210.50)	< 0.001
Potassium (mmol/L) (median (IQR))	4.10 (3.80, 4.50)	4.13 (3.75, 4.60)	4.10 (3.78, 4.48)	0.264
Sodium (mmol/L) (median (IQR))	138.50 (135.13, 141.71)	138.67 (135.35, 141.67)	138.90 (135.00, 141.46)	0.719
Calcium (mmol/L) (median (IQR))	8.10 (7.60, 8.60)	7.90 (7.47, 8.43)	8.00 (7.52, 8.63)	0.009
Chloride (mmol/L) (median (IQR))	104.00 (100.00, 108.00)	105.00 (100.50, 109.00)	104.00 (99.50, 108.73)	0.067
Aniongap (mmol/L) (median (IQR))	14.00 (12.00, 16.78)	15.10 (12.67, 18.00)	16.00 (13.00, 19.50)	< 0.001
Bicarbonate (mmol/L) (median (IQR))	22.33 (19.67, 24.50)	21.00 (18.00, 24.00)	21.25 (17.56, 24.19)	< 0.001
PT (s) (median (IQR))	15.50 (13.30, 18.05)	16.00 (13.65, 19.39)	17.08 (14.31, 20.326)	< 0.001
PTT (s) (median (IQR))	33.30 (28.80, 42.30)	35.60 (30.01, 45.72)	36.84 (30.52, 46.37)	0.001
INR (median (IQR))	1.40 (1.20, 1.65)	1.45 (1.21, 1.80)	1.55 (1.30, 1.89)	< 0.001
PH (median (IQR))	7.38 (7.33, 7.42)	7.36 (7.30, 7.41)	7.35 (7.29, 7.40)	< 0.001
Lactate (mmol/L) (median (IQR))	1.77 (1.24, 2.43)	2.18 (1.40, 3.53)	2.58 (1.58, 4.40)	< 0.001
HR (beats/minute) (median (IQR))	92.30 (78.70, 103.92)	90.06 (77.48, 103.43)	91.23 (81.22, 103.57)	0.535
SBP (mmHg) (median (IQR))	110.73 (102.67, 122.17)	109.46 (101.77, 117.49)	108.25 (100.25, 120.91)	0.108
DBP (mmHg) (median (IQR))	61.58 (55.80, 68.63)	60.84 (55.13, 67.08)	61.42 (55.31, 68.52)	0.214
MBP (mmHg) (median (IQR))	75.83 (70.12, 82.98)	74.83 (70.13, 81.24)	75.95 (69.33, 82.95)	0.470
Temperature (°C) (median (IQR))	37.01 (36.71, 37.45)	36.88 (36.61, 37.29)	36.81 (36.57, 37.13)	< 0.001
SPO₂ (%) (median (IQR))	97.23 (95.65, 98.56)	97.30 (95.58, 98.74)	97.57 (95.85, 98.84)	0.373
RR (beats/minute) (median (IQR))	19.92 (17.49, 23.05)	20.39 (17.60, 24.07)	20.40 (17.34, 23.39)	0.375
GCS (median (IQR))	15.00 (14.33, 15.00)	15.00 (14.67, 15.00)	15.00 (14.39, 15.00)	0.039
SAPS II (median (IQR))	54.00 (42.00, 68.00)	58.00 (45.00, 74.00)	60.50 (48.00, 79.00)	< 0.001
SOFA (median (IQR))	4.00 (2.00, 5.00)	4.00 (3.00, 6.00)	4.00 (3.00, 7.00)	< 0.001
Lymphocytes (10^9/L) (median (IQR))	0.76 (0.21, 1.54)	0.78 (0.30, 1.57)	0.72 (0.16, 1.53)	0.443
Monocytes (10^9/L) (median (IQR))	0.67 (0.44, 0.93)	0.64 (0.41, 0.90)	0.59 (0.32, 0.85)	0.042
Neutrophils (10^9/L) (median (IQR))	10.33 (8.07, 13.20)	11.04 (7.86, 14.59)	10.73 (8.46, 14.30)	0.080
NLR (median (IQR))	15.49 (10.25, 19.95)	16.08 (9.94, 20.88)	15.82 (9.93, 21.54)	0.455
PLR (median (IQR))	242.61 (144.45, 380.53)	219.85 (131.38, 354.94)	192.54 (116.75, 358.18)	0.047
SII (median (IQR))	2069.62 (992.89, 4134.23)	2176.76 (927.43, 4198.86)	1787.50 (900.69, 4360.52)	0.903
Outcomes				
28‐day mortality *n* (%)	83 (15.81%)	112 (17.28%)	43 (23.89%)	0.047
180‐day mortality *n* (%)	165 (31.43%)	213 (32.87%)	73 (40.56%)	0.048

Abbreviations: BUN, blood urea nitrogen; DBP, diastolic blood pressure; GCS, Glasgow coma scale; GVC, glycemic variability coefficient; HR, heart rate; INR, international normalized ratio; IQR, interquartile range; MBP, mean blood pressure; NLR, neutrophil‐to‐lymphocyte ratio; PH, potential of hydrogen; PLR, platelet‐to‐lymphocyte ratio; PT, prothrombin time; PTT, partial thromboplastin time; RBC, red blood cells; RR, respiratory rate; SAPS II, simplified acute physiology score II; SBP, systolic blood pressure; SD, standard deviation; SII, systemic immune‐inflammation index; SOFA, sequential organ failure assessment; SPO2, saturation of peripheral oxygen; WBC, white blood cells.

### 3.2 Clinical Outcomes

**Figure 2:**
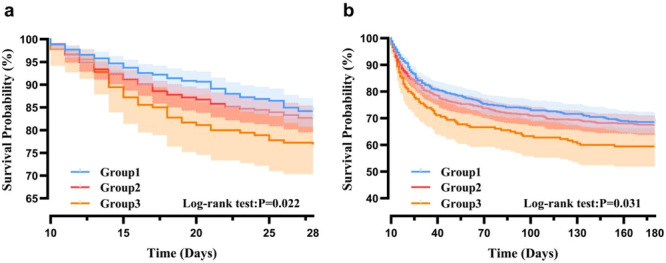
Kaplan–Meier survival curves for sepsis patients with PICS stratified by GVC levels. (a) 28‐day survival curve; (b) 180‐day survival curve. Legends: Group 1 (GVC < 20, *n* = 525), Group 2 (20 ≤ GVC ≤ 36, *n* = 648), and Group 3 (GVC > 36, *n* = 180). Statistical method: Log‐rank test for comparison of survival differences between groups.

Group 3 (highest‐GVC cohort) exhibited significantly higher 28‐day and 180‐day mortality rates compared to other groups (Table 1). To further test our hypothesis, we analyzed the association between GVC and mortality via Kaplan–Meier and Cox regression. The Kaplan–Meier curve results indicated that the third group had the lowest survival probabilities at both 28 days and 180 days, with the differences being statistically significant (Figure 2). Unadjusted Cox regression analysis demonstrated that, using Quartile Group 1 (lowest GVC group) as reference, Quartile Group 3 exhibited significantly elevated mortality risk (28‐day mortality: OR = 1.60, 95% CI: 1.11–2.32, *p* < 0.05; in‐hospital mortality: OR = 1.40, 95% CI: 0.98–2.01, *p* > 0.05; 180‐day mortality: OR = 1.42, 95% CI: 1.07–1.86, *p* < 0.05) (Table 2). After adjustment for age, weight, sex, vital signs, SOFA score, and lymphocyte count, this trend remained consistent (Model II, *p* < 0.05).

**Table 2:** Cox regression model (in‐hospital mortality, 28‐day mortality, and 180‐day mortality).

Model	Group	In‐hospital mortality		28‐day mortality		180‐day mortality	
		*p*‐value	Result	*p*‐value	Result	*p*‐value	Result
Model 1	Group 1		1 (Reference)		1 (Reference)		1 (Reference)
	Group 2	0.413	1.12 (0.86, 1.46)	0.437	1.12 (0.84, 1.49)	0.586	1.06 (0.86, 1.30)
	Group 3	0.065	1.40 (0.98, 2.01)	0.012	1.60 (1.11, 2.32)	0.014	1.42 (1.07, 1.86)
Model 2							
	Group 1		1 (Reference)		1 (Reference)		1 (Reference)
	Group 2	0.796	1.04 (0.79, 1.36)	0.880	1.02 (0.77, 1.36)	0.881	0.98 (0.73, 1.31)
	Group 3	0.093	1.37 (0.95, 1.98)	0.038	1.49 (1.02, 2.17)	0.263	1.38 (0.78, 2.44)

Model 1 represents the unadjusted Cox regression analysis, while Model 2 is adjusted for age, weight, gender, heart rate, respiratory rate, systolic blood pressure, SOFA score, and absolute lymphocyte count.

### 3.3 Boruta Algorithm

**Figure 3:**
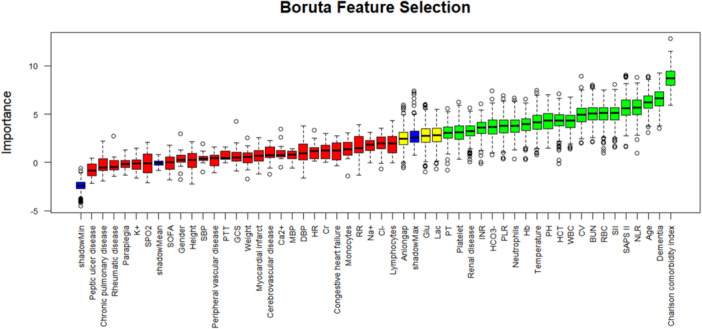
Feature selection based on the Boruta algorithm.

To verify whether GVC is a key predictor of mortality, Boruta algorithm was performed for feature selection, as shown in Figure 3, which evaluated feature importance by calculating the Z‐scores of variables and comparing them with the threshold of randomly generated “shadow features.” The horizontal axis of the figure displays variable names, while the vertical axis represents the Z‐scores of variables during model computation. The boxplot visually presents the distribution characteristics of Z‐scores for each variable. Among these, green boxes indicate important variables and red boxes denote non‐important variables. The horizontal axis displays variable names, while the vertical axis represents Z‐scores from model computation. Boxplots visually demonstrate the distribution characteristics of Z‐scores across variables, with green boxes indicating important variables, including Charlson comorbidity index, NLR, SII, SAPS II, RBC, WBC, Platelet, HCT, GVC, PH, PLR, and Hb, while red boxes denoting non‐important variables. GVC ranked among the top 10 significant predictors in this study.

### 3.4 Model Performance Comparison

**Figure 4:**
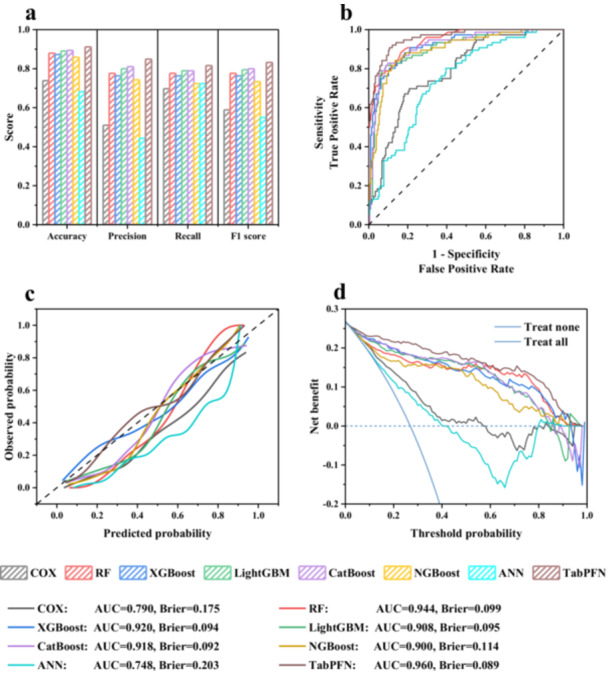
Prediction results of eight ML/DL models were evaluated using (a) accuracy, precision, recall, and F1 index, (b) ROC, (c) calibration curve, and (d) DCA.

To further validate the predictive value of GVC, ML/DL models were employed to enable more robust pattern recognition, thereby enhancing clinical practice. The key features selected by the Boruta algorithm were employed for ML/DL modeling. Figure 4a indicates that TabPFN outperformed other models with an accuracy of 0.912, precision of 0.849, recall of 0.816, and F1 score of 0.832. Meanwhile, TabPFN demonstrated the best discrimination with AUC of 0.960 (Figure 4b). The calibration curves show that the model had better calibration across the spectrum of survival probabilities, with the lowest Brier score of 0.137 (Figure 4c). Furthermore, Figure 4d shows a strong positive net benefit of TabPFN across a wide threshold range, indicating that the established model has robust clinical validity.

### 3.5 Model Interpretation

**Figure 5:**
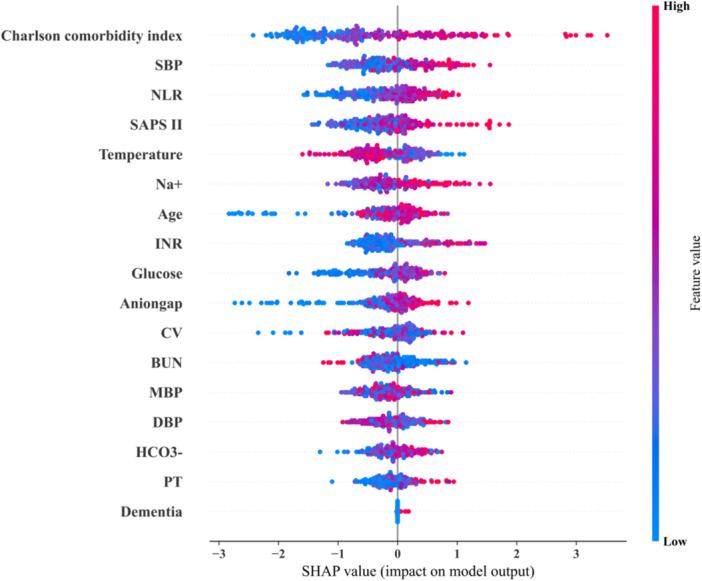
SHAP plot used to interpret the prediction mechanism of TabPFN.

The contribution of each feature to the death risk prediction in TabPFN was evaluated using SHAP method (Figure 5). On the vertical axis, each feature was ranked according to the mean |SHAP| value of each feature across all samples, which indicates the global importance of each feature; on the horizontal axis, the SHAP value for each sample is shown, which represents the distribution of the influence of features on the model output. Colors denote feature values (red for high and blue for low), which illustrate the impact of feature variation on outcomes. The result indicates that GVC was found to be critical for TabPFN.

### 3.6 External Validation

**Figure 6:**
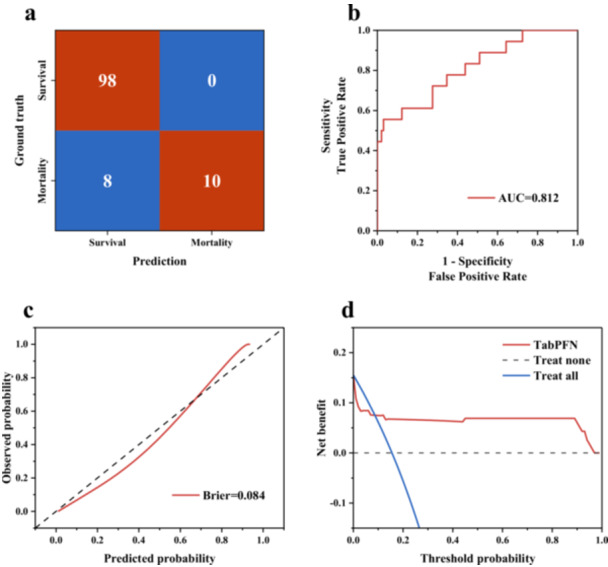
External validation results of the TabPFN were evaluated using (a) confusion matrix, (b) ROC, (c) calibration curve, and (d) DCA.

To confirm the generalizability of the hypothesis across diverse populations, the external validation using 116 sepsis patients with concurrent PICS from the Emergency Department of Tianjin Medical University General Hospital was performed. The inclusion and exclusion criteria for the external validation cohort were consistent with those of the training cohort, ensuring comparability in patient characteristics. Figure 6a shows that the TabPFN model maintained robust predictive performance in the external validation cohort. Figure 6b demonstrates a powerful discrimination ability of the model for 28‐day survivors and non‐survivors with AUC of 0.812, while Figure 6c reveals that the predicted probabilities of 28‐day mortality were closely aligned with the observed probabilities with Brier score of 0.084, confirming the strong discrimination and calibration of TabPFN across diverse clinical settings and patient populations. DCA shows that when the threshold probability ranged from 0.1 to 0.8, the net benefit of the TabPFN model was significantly higher than the “treat all” and “treat none” strategies, indicating that applying the model to guide risk stratification in clinical practice could bring meaningful clinical gains. The external validation results confirmed that the TabPFN maintained stable predictive performance in a geographically and institutionally distinct cohort, which supports the potential for clinical translation as a tool for risk assessment in sepsis patients with PICS.

## 4 Discussion

This study systematically investigated the association between GVC and clinical outcomes in sepsis patient subgroups developing PICS. After adjusting for age, sex, SOFA score, and other confounders, increasing GVC corresponded to elevated 28‐day and 180‐day mortality rates, with this association demonstrating consistent strength across both time points. The robustness of this study is evidenced through multi‐dimensional validation.

The Boruta algorithm demonstrated the significant importance of GVC among 54 candidate indicators. In addition, Cox proportional hazards regression and SHAP analysis demonstrated the substantial contributions of GVC. The TabPFN model further provided algorithmic‐level support for the clinical translation of GVC with AUC of 0.960 and Brier score of 0.089 in the internal validation and AUC of 0.812 and Brier score of 0.084 in the external validation, demonstrating the prognostic discrimination efficacy of GVC‐related parameters. These results confirm the critical role of GVC in dynamic glycemic assessment and extend the clinical significance of GVC management in the PICS‐afflicted sepsis subpopulation, demonstrating its potential value for clinical decision‐making and patient management strategies targeting this specific cohort.

Existing evidence established GVC as an independent predictor of microvascular complications in diabetes management, with longitudinal studies confirming its specific association with heightened diabetic retinopathy risk [17]. In non‐diabetic critical illnesses, GVC demonstrates a J‐curve relationship with cardiogenic shock incidence among acute myocardial infarction patients, indicating its disease‐specific pathophysiological relevance [32, 33, 34]. Preliminary observational evidence suggests GVC may serve as a candidate marker for prognostic stratification, disordered metabolic states, and atherosclerosis progression in critically ill populations [33, 34, 35]. Glycemic fluctuations may reflect sepsis‐specific homeostatic collapse mechanisms. Disordered metabolism plays a postulated role in sepsis progression, with concurrent associations observed between elevated GVC and both metabolic acidosis development and chronic inflammatory states during the disease course [36, 37]. GVC modulation potentially influences cellular nutrient utilization and energy production. Clinical observations indicate that elevated GVC in sepsis patients correlates with inflammatory response activation and altered cellular energetics, which are linked to increased mortality risk and prolonged hospitalizations [38, 39]. Within the sepsis population, glycemic variability demonstrates a close association with clinical outcomes. Both sustained hyperglycemia and its fluctuation magnitude are independently associated with increased mortality risk in these patients [40, 41]. Elevated GVC signifies clinical instability during illness fluctuation, exerting a substantive impact on sepsis management. Studies demonstrate that GVC increases correlate with aggravated multi‐organ dysfunction syndrome (MODS) in septic patients, which is mechanistically plausible through systemic metabolic derangement and endocrine response dysregulation [17]. Evidence indicates standard deviation and GVC of glucose levels predict disseminated intravascular coagulation (DIC) risk in this population, underscoring the imperative for glucose stability management during critical illness progression [17, 41]. Accumulating evidence confirms that GVC demonstrates significant associations with clinical outcomes in critical illnesses and infectious conditions.

Studies analyzing glycemic fluctuations during the first week post‐admission demonstrate that sepsis patients with higher glucose variability levels manifest elevated propensity for PICS. This substantiates the imperative for enhanced GVC monitoring and control in clinical management protocols [42]. Elevated GVC is postulated to directly impair lymphocyte function via oxidative stress and mitochondrial dysfunction. Concurrently, pronounced glucose excursions may exacerbate endoplasmic reticulum stress, collaboratively driving functional exhaustion in immune cells [12]. High levels of GVC correlate with increased expression of multiple inflammatory mediators, demonstrably contributing to immune cell overstimulation and inflammatory amplification. Glucose excursions stimulate NADPH oxidase activity, generating reactive oxygen species (ROS) that activate NF‐κB signaling and upregulate IL‐6/TNF‐α [12]. Glycemic variability not only impacts immune responses but also potentiates protein catabolism and muscle wasting through interference with insulin signaling pathways and energy metabolism. This pathophysiological process demonstrates direct relevance to the catabolic phenotype characteristic of PICS. GVC fluctuations demonstrate association with exacerbated insulin resistance, thereby disrupting systemic metabolic equilibrium [43]. These mechanisms indicate that GVC contributes to the pathophysiological regulation of PICS in septic patients through complex immunometabolic interactions. Concurrent evidence confirms this dysregulation independently correlates with increased mortality risk.

The present analysis confirms that GVC maintains significant prognostic value in patients without preexisting diabetes. While GVC ranked 11th in SHAP value importance among predictors, its status as a core prognostic covariate is substantiated through multidimensional evidence. SHAP values reflect the relative contribution of features within the model, influenced by algorithmic characteristics and variable interactions. As a nonlinear derived metric, the complex mechanism of GVC action cannot be fully captured by a single model weight. The Boruta algorithm categorically classified GVC as a “significant feature” (Z‐score surpassing shadow feature thresholds). Survival analyses substantiate that the highest‐GVC group exhibits significantly elevated 28‐day and 180‐day mortality rates compared to those with stable glucose profiles, with covariate‐adjusted odds ratios maintaining statistical significance. This prognostic pattern likely reflects the integration of both short‐term glycemic excursions and chronic metabolic baselines. Elevated GVC may synergistically potentiate insulin resistance, amplified oxidative stress, and immune cell functional suppression during PICS states, collectively exacerbating risks of dysregulated infection responses and organ injury, ultimately driving worsened long‐term outcomes in septic PICS patients [44, 45, 46].

This investigation harbors inherent methodological constraints. As a retrospective analysis utilizing preexisting records, this study may harbor residual confounding from unmeasured variables. Second, partial study data originated from 2008 to 2019 databases, during which temporal evolution of treatment strategies may have exerted a quantifiable impact on outcome measurements. Third, the geographically constrained sample size in the external validation cohort necessitates confirmation of model generalizability through multicenter investigations with enhanced statistical power. Finally, this research primarily established associations between baseline glycemic variability and clinical outcomes. The temporal dynamics of in‐hospital glycemic variation and dynamic PICS evolution remain insufficiently explored. Future research should employ more advanced methods to dissect the heterogeneity within PICS, for example, by inflammation vs. catabolism dominance.

## 5 Conclusion

Glycemic coefficient of variation demonstrates significant associations with both 28‐day and 180‐day mortality rates in critically ill sepsis patients developing persistent inflammation, immunosuppression, and catabolism syndrome. Elevated GVC levels correlate substantially with increased risk of adverse clinical outcomes, establishing GVC as a potential predictor for mid‐to‐long‐term adverse outcomes in this patient subpopulation.

## Author Contributions

**Shuhang Wang:** writing – original draft, writing – review and editing. **Li Liu** and **Bowen Li:** writing – review and editing, data curation, formal analysis, methodology. **Yancun Liu** and **Yanfen Chai:** writing – review and editing, funding acquisition.

## Ethics Statement

The authors affirm that our study adhered to the Declaration of Helsinki and we followed professional ethical guidelines when preparing this work. These guidelines include maintaining ethical treatment and respect for the rights of human participants and ensuring the privacy of participants and their data, such as ensuring that individual participants cannot be identified in reported results or from publicly available original or archival data. This study was approved by the Scientific Investigation Ethical Review Board of Tianjin Medical University General Hospital (No. IRB2021‐YX‐220‐01), which waived the requirement for informed patient consent because of the retrospective nature of the analysis.

## Conflicts of Interest

The authors declare no conflicts of interest.

## Supporting information


**Figure S1:** Schoenfeld residuals to rigorously test the proportional hazards hypothesis of the Cox model: (A‐C) Modle1; (D‐F) Modle2. **Table S1:** Hyperparameter tuning of each ML model. **Table S2:** Baseline characteristics of patients in the emergency department of Tianjin Medical University General Hospital.

## Data Availability

The data that support the findings of this study are available from the corresponding author upon reasonable request.
