# Supplementary material for: Glycemic Variability as a Predictor of Mortality in Sepsis Patients With Concurrent Persistent Inflammation, Immunosuppression, and Catabolism Syndrome
Source: Immun Inflamm Dis. 2026 Mar 6;14(3):e70400. doi: 10.1002/iid3.70400 (PMC12965729; doi:10.1002/iid3.70400)
Supplement: Supplementary file 1 — Figure S1: Schoenfeld residuals to rigorously test the proportional hazards hypothesis of the Cox model: (A‐C) Modle1; (D‐F) Modle2. Table S1: Hyperparameter tuning of each ML model. Table S2: Baseline characteristics of patients in the emergency department of Tianjin Medical University General Hospital. [file IID3-14-e70400-s001.docx]

Supporting Information

Glycemic Variability as a Predictor of Mortality in Sepsis Patients with Concurrent Persistent Inflammation, Immunosuppression, and Catabolism Syndrome

Shuhang Wang^1†^, Li Liu^1†^, Bowen Li^2†^, Yancun Liu^1*^, Yanfen Chai^1*^

*^1^ Tianjin Medical University General Hospital, Tianjin 300052, China*

*^2^ College of Environmental Science and Engineering, Nankai University, Tianjin 300350, China*

* *Corresponding author:*

*Yancun Liu, E-mail address: yancunliu@tmu.edu.cn; Yanfen Chai, E-mail address: [chaiyanfen2012@126.com](mailto:chaiyanfen2012@126.com)*

*First authors:*

*Shuhang Wang, E-mail address: wgshu33@gmail.com; Li Liu, E-mail address: [liliu0504@tmu.edu.cn;](mailto:liliu0504@tmu.edu.cn;) Bowen Li, E-mail address: 593160536@qq.com*

†：These authors contributed equally.


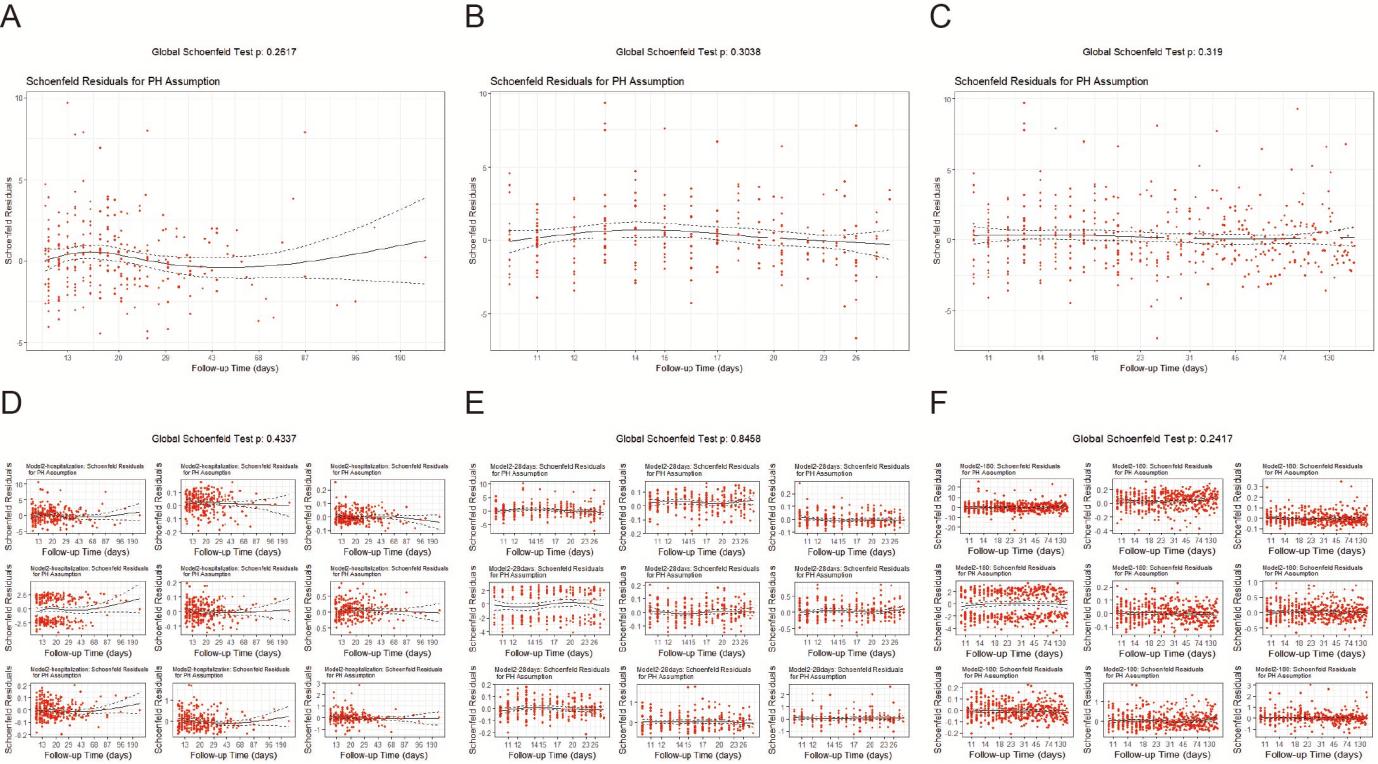


**Fig. S1.** Schoenfeld residuals to rigorously test the proportional hazards hypothesis of the Cox model: (A-C) Modle1; (D-F) Modle2.

**Table S1** Hyperparameter tuning of each ML model.

| Model | Hyperparameter | Value |
| --- | --- | --- |
| COX | Penalizer | 0.02 |
|  | L1 ratio | 1.0 |
| RF | Number of trees | 300 |
|  | Minimum samples in node splitting | 10 |
|  | Minimum samples at leaf node | 5 |
| XGBoost | Maximum depth of a tree | 6 |
|  | Minimum sum of instance weight needed in a leaf | 5 |
|  | Subsample ratio of the training instances | 0.8 |
| LightGBM | Number of boosted trees | 300 |
|  | Learning rate | 0.05 |
|  | Minimum sum of instance weight needed in a leaf | 8 |
|  | Subsample ratio of the training instances | 0.8 |
| CatBoost | Number of trees | 350 |
|  | Learning rate | 0.05 |
|  | Maximum depth of a tree | 7 |
|  | Subsample ratio of the training instances | 0.8 |
|  | Random strength | 1.0 |
|  | Bagging temperature | 0.7 |
| NGBoost | Learning rate | 0.05 |
|  | Minibatch fraction | 0.8 |
|  | Column subsampling | 0.5 |
| ANN | Learning rate | 0.001 |
|  | Batch size | 32 |
|  | Number of hidden layers | 2 |
|  | Number of units in each hidden layer | 128 |
|  | Dropout ratio | 0.2 |
| TabPFN | Using the default setting^1, 2^ |  |

**Table S2** Baseline characteristics of patients in the emergency department of Tianjin Medical University General Hospital.

| Features | Group 1 (GVC<20) | Group 2 (20≤GVC≤36) | Group 3 (GVC>36) | p-value |
| --- | --- | --- | --- | --- |
| **Demographic** |  |  |  |  |
| Gender (male) (n%) | 319 (60.76) | 357 (55.09) | 97 (53.89) | 0.096 |
| Hight (cm) (median (IQR)) | 171.985 (163, 178) | 168 (161, 175) | 168 (160, 175) | 0.003 |
| Weight (kg) (median (IQR)) | 79.3 (67.05, 98) | 78.3 (67.73, 95.4) | 73.275 (60, 88.83) | <0.001 |
| Age (years) (median (IQR)) | 61.84 (48.73, 74.40) | 64.13 (53.47, 75.20) | 62.37 (51.69, 75.05) | 0.062 |
| **Comorbidities** |  |  |  |  |
| Myocardial infarct (n%) | 11 (15.2) | 7 (18.7) | 3 (23.1) | 0.067 |
| Congestive heart failure (n%) | 17 (23.5) | 9 (27.8) | 4 (32.6) | 0.023 |
| Peripheral vascular disease (n%) | 9 (12.8) | 5 (16.3) | 2 (15.4) | 0.089 |
| Cerebrovascular disease (n%) | 13 (18.5) | 7 (21.1) | 4 (30.7) | 0.041 |
| Dementia (n%) | 6 (8.5) | 4 (12.1) | 2 (15.3) | 0.035 |
| Chronic pulmonary disease (n%) | 10 (14.29) | 1 (3.03) | 0 (0) | 0.091 |
| Rheumatic disease (n%) | 1 (1.43) | 1 (3.03) | 3 (23.07) | 0.002 |
| Peptic ulcer disease (n%) | 5 (7.14) | 3 (6.06) | 1 (7.69) | 0.973 |
| Renal disease (n%) | 24 (34.28) | 5 (15.15) | 5 (38.46) | 0.163 |
| Charlson comorbidity index (median (IQR)) | 2(1,3) | 1(0,3) | 3(1,4) | 0.326 |
| **Clinical indicators** |  |  |  |  |
| Hemoglobin (g/dL) (median (IQR)) | 11.20 (9.72, 12.17) | 10.60 (8.60, 12.70) | 9.70 (8.80, 10.90) | 0.381 |
| Hematocrit (median (IQR)) | 33.35 (29.42, 36.33) | 32.70 (25.90, 37.60) | 29.70 (27.90, 34.70) | 0.471 |
| Platelet (10^9/L) (median (IQR)) | 159.50 (102.50, 201.25) | 163.00(115.00, 239.00) | 111.00(90.00, 182.00) | 0.441 |
| Wbc (10^9/L) (median (IQR)) | 9.68 (6.68, 14.59) | 12.03 (6.99, 21.35) | 10.68 (8.83, 13.26) | 0.309 |
| Rbc (10^12/L) (median (IQR)) | 3.62 (3.03, 4.06) | 3.74 (3.00, 4.04) | 2.93 (2.85, 3.79) | 0.431 |
| Creatinine (mg/dL) (median (IQR)) | 0.97 (0.69, 1.91) | 0.80 (0.60, 1.81) | 0.93 (0.76, 2.06) | 0.587 |
| BUN (mg/dL) (median (IQR)) | 10.15 (7.15, 15.40) | 8.31 (6.10, 15.70) | 27.00 (13.08, 43.63) | 0.626 |
| Glucose (mg/dL) (median (IQR)) | 7.70 (6.00, 9.20) | 6.70 (5.70, 12.10) | 140.00 (111.50, 210.50) | 0.003 |
| Potassium (mmol/L) (median (IQR)) | 3.49 (3.10, 4.00) | 3.37 (3.06, 3.87) | 3.60 (3.35, 3.81) | 0.622 |
| Sodium (mmol/L) (mean (SD)) | 134.86 ± 8.31 | 136.49 ± 5.83 | 140.55 ± 10.10 | 0.056 |
| Calcium (mmol/L) (median (IQR)) | 1.02(0.96,1.06) | 1.02(0.98,1.10) | 1.07(1.04,1.11) | 0.097 |
| Chloride (mmol/L) (mean (SD)) | 107.22 ± 8.38 | 108.18 ± 6.15 | 110.68 ± 10.55 | 0.358 |
| Aniongap (mmol/L) (median (IQR)) | 10.30(7.65,14.40) | 7.30(5.95,10.53) | 8.80(7.50,14.10) | 0.031 |
| Bicarbonate (mmol/L) (median (IQR)) | 21.05(17.85,23.25) | 24.00(19.60,27.00) | 24.20(18.60,25.70) | 0.043 |
| PT (s) (median (IQR)) | 12.55(11.30,13.90) | 12.80(11.30,15.00) | 13.50(12.05,18.40) | 0.460 |
| PTT (s) (median (IQR)) | 33.85(29.35,42.60) | 31.30(27.60,38.40) | 31.40(28.43,40.48) | 0.453 |
| INR (median (IQR)) | 1.14(1.03,1.27) | 1.14(1.03,1.38) | 1.25(1.07,1.70) | 0.416 |
| PH (median (IQR)) | 7.41(7.35,7.46) | 7.42(7.37,7.45) | 7.44(7.37,7.45) | 0.693 |
| Lactate (mmol/L) (median (IQR)) | 1.60(1.20,2.10) | 1.80(1.50,2.28) | 1.60(1.50,2.80) | 0.346 |
| HR (median (IQR)) | 92.30 (78.70, 103.92) | 90.06 (77.48, 103.43) | 91.23 (81.22, 103.57) | 0.535 |
| SBP (mmHg) (median (IQR)) | 110.73 (102.67, 122.17) | 109.46 (101.77, 117.49) | 108.25 (100.25, 120.91) | 0.108 |
| DBP (mmHg) (median (IQR)) | 61.58 (55.80, 68.63) | 60.84 (55.13, 67.08) | 61.42 (55.31, 68.52) | 0.214 |
| MBP (mmHg) (median (IQR)) | 75.83 (70.12, 82.98) | 74.83 (70.13, 81.24) | 75.95 (69.33, 82.95) | 0.471 |
| Temperature (℃) (median (IQR)) | 37.01 (36.71, 37.45) | 36.88 (36.61, 37.29) | 36.81 (36.57, 37.13) | 0.001 |
| SPO₂ (%) (median (IQR)) | 97.23 (95.65, 98.56) | 97.30 (95.58, 98.74) | 97.57 (95.85, 98.84) | 0.373 |
| RR (median (IQR)) | 19.92 (17.49, 23.05) | 20.39 (17.60, 24.07) | 20.40 (17.34, 23.39) | 0.375 |
| GCS (median (IQR)) | 13.00(7.00,14.00) | 13.00(10.00,14.00) | 7.00(4.00,13.00) | 0.033 |
| SAPS II (median (IQR)) | 36.00(29.25,45.75) | 39.00(30.00,49.00) | 39.00(34.00,58.00) | 0.421 |
| SOFA (median (IQR)) | 6.00(3.25,7.75) | 5.50(2.75,7.25) | 8.00(7.00,12.00) | 0.015 |
| Lymphocytes (10^9/L) (median (IQR)) | 0.81(0.58,1.30) | 0.78(0.56,1.28) | 0.87(0.51,1.18) | 0.768 |
| Monocytes (10^9/L) (median (IQR)) | 0.63(0.41,0.81) | 0.51(0.33,0.92) | 0.43(0.35,0.79) | 0.541 |
| Neutrophils (10^9/L) (median (IQR)) | 7.72(4.83,12.47) | 9.57(4.91,19.62) | 10.52(7.81,12.02) | 0.207 |
| NLR (median (IQR)) | 5.89(0.98,11.20) | 12.07(3.32,22.47) | 13.82(7.28,24.22) | 0.001 |
| PLR (median (IQR)) | 186.42(113.60,299.06) | 164.68(108.33,330.46) | 186.27(128.30,248.31) | 0.866 |
| SII (median (IQR)) | 1373.36(671.23,2601.08) | 1827.57(746.25,3511.08) | 1837.54(953.71,2712.35) | 0.335 |
| **Outcomes** |  |  |  |  |
| 28-Day Mortality (n%) | 3 (4.28) | 5 (15.15) | 9 (69.23) | <0.001 |
| 180-Day Mortality (n%) | 19 (27.15) | 13 (39.39) | 10 (76.92) | <0.001 |

# References

1. Hollmann NM, Samuel, Eggensperger K, Hutter F. TabPFN: A transformer that solves small tabular classification problems in a second. presented at: International Conference on Learning Representations 2023; 2023;

2. Hollmann N, Müller S, Purucker L, et al. Accurate predictions on small data with a tabular foundation model. *Nature*. 2025/01/01 2025;637(8045):319-326. doi:10.1038/s41586-024-08328-6
